# Plant-Based Diets and Their Role in Preventive Medicine: A Systematic Review of Evidence-Based Insights for Reducing Disease Risk

**DOI:** 10.7759/cureus.78629

**Published:** 2025-02-06

**Authors:** Sultan A Almuntashiri, Faris F Alsubaie, Moteab Alotaybi

**Affiliations:** 1 Department of Family and Community Medicine, King Abdulaziz Medical City, Jeddah, SAU; 2 Department of Family Medicine, Makkah Cluster of Health, Ministry of Health, Makkah, SAU; 3 Department of Preventive Medicine, Ministry of National Guard, Jeddah, SAU

**Keywords:** cardiovascular health, chronic disease prevention, dietary interventions, gut microbiome, inflammation, metabolic health, personalized nutrition, plant-based diets, preventive medicine, weight management

## Abstract

Plant-based diets have gained increasing attention for their potential role in preventive medicine, particularly in reducing the risk of chronic diseases such as type 2 diabetes, cardiovascular disease, obesity, and metabolic syndrome. This systematic review synthesizes evidence from 32 longitudinal studies to evaluate the impact of plant-based diets on disease prevention and health outcomes. The review identifies consistent patterns, including improved metabolic health, weight management, cardiovascular risk reduction, and positive effects on gut microbiome composition and inflammation. However, inconsistencies arise due to variability in diet definitions, mixed findings on specific outcomes, and heterogeneity in study populations. Critical gaps in the literature include the lack of long-term studies, limited mechanistic insights, underrepresentation of diverse populations, and a need for more rigorous intervention studies and personalized nutrition approaches. Identified research gaps highlight the need for long-term studies, deeper exploration of mechanistic pathways, and greater inclusivity of diverse populations. These insights underscore the significance of plant-based diets as a cornerstone of preventive medicine while emphasizing the necessity for targeted interventions and personalized approaches to maximize their benefits. The findings contribute to a growing body of evidence supporting the integration of plant-based dietary strategies into public health policies and clinical practices.

## Introduction and background

Plant-based diets, characterized by a high intake of fruits, vegetables, legumes, whole grains, nuts, and seeds, with minimal or no animal products, have gained significant attention in recent years for their potential health benefits [1,2]. With the global rise of chronic diseases like cardiovascular disease, type 2 diabetes, obesity, and certain cancers, there is increasing interest in dietary patterns that help reduce these risks [3,4]. Plant-based diets have emerged as a promising strategy in preventive medicine, supported by a growing body of evidence suggesting their role in reducing disease risk and promoting overall health [5].

The shift toward plant-based eating is rooted in the understanding that diet is a modifiable risk factor for many chronic conditions. Epidemiological studies have consistently shown that populations consuming predominantly plant-based diets, such as those in the Mediterranean region or adhering to traditional Asian diets, exhibit lower rates of chronic diseases and longer life expectancy [6,7]. These diets are rich in fiber, antioxidants, phytochemicals, and healthy fats, which collectively contribute to their protective effects [8]. In contrast, diets high in processed meats, saturated fats, and refined sugars have been linked to increased inflammation, oxidative stress, and metabolic dysfunction, all of which are precursors to chronic disease [9].

One of the most compelling areas of research on plant-based diets is their impact on cardiovascular health. Research has demonstrated that plant-based diets can lower blood pressure, improve lipid profiles, and reduce the risk of coronary artery disease [10]. The high fiber content of plant-based diets plays a crucial role in reducing cholesterol levels, while the abundance of potassium and magnesium helps regulate blood pressure. Additionally, the anti-inflammatory properties of plant foods contribute to improved endothelial function and reduced arterial stiffness [2,11].

Plant-based diets also help in the prevention and management of type 2 diabetes. Research indicates that these diets improve insulin sensitivity, reduce HbA1c levels, and lower the risk of developing diabetes by up to 34% [12]. The low glycemic load of plant-based foods, combined with their high nutrient density, helps stabilize blood sugar levels and prevent insulin resistance [13,14]. Furthermore, plant-based diets are associated with healthier body weight, which is a critical factor in diabetes prevention [14].

Emerging evidence also highlights the role of plant-based diets in cancer prevention, as phytochemicals such as flavonoids, carotenoids, and glucosinolates, found abundantly in plant foods, have been shown to possess anti-carcinogenic properties [15]. These compounds help neutralize free radicals, reduce DNA damage, and inhibit the growth of cancer cells [16].

While more research is needed to establish definitive causal relationships, the protective effects of plant-based diets against certain cancers, particularly colorectal and breast cancer, are increasingly recognized [17].

Despite the growing evidence of the benefits of the plant-based diet, challenges remain in promoting the widespread adoption of plant-based diets. Misconceptions about nutrient adequacy, particularly regarding protein, iron, and vitamin B12, often deter individuals from transitioning to plant-based eating. However, with proper planning and education, plant-based diets can meet all nutritional requirements and offer a sustainable approach to disease prevention [18]. This indicates the importance of bridging the gap between scientific evidence and practical applications in preventive medicine.

The rationale for this systematic review is grounded in the urgent need to address the global burden of chronic diseases through evidence-based dietary interventions. By synthesizing the existing research, this review aims to provide a clear, comprehensive, and actionable understanding of how plant-based diets can reduce disease risk, ultimately contributing to improved public health outcomes. Therefore, this review aimed to synthesize evidence from available literature, exploring the role of plant-based diets and reduced risk of chronic diseases and identifying the mechanisms through which plant-based diets exert their preventive effects, with variations in outcomes and in diverse populations.

## Review

Methods

Design

This was a systematic review of longitudinal studies. The primary research question guiding this review was as follows: What is the role of plant-based diets in preventive medicine, and how do they influence the risk of chronic diseases such as type 2 diabetes, cardiovascular disease, obesity, and metabolic syndrome?

Search Strategy

A comprehensive and systematic search strategy was developed to identify relevant studies. The search was conducted across multiple electronic databases: PubMed, Scopus, Web of Science, and Cochrane Library. The search terms were tailored to capture studies related to plant-based diets and their health outcomes and Boolean operators (AND, OR) to optimize the search results. These combinations were used: ("plant-based diet" OR "vegetarian diet" OR "vegan diet" OR "Mediterranean diet") AND ("chronic disease" OR "diabetes" OR "cardiovascular disease" OR "obesity" OR "metabolic syndrome") AND ("prevention" OR "risk reduction" OR "health outcomes").

The search was limited to studies published between 2013 and 2023 to ensure the inclusion of recent evidence. Additionally, reference lists of included studies were manually searched to identify additional relevant articles.

Eligibility Criteria

We included only longitudinal and interventional studies published in peer-reviewed journals involving human subjects and studies that explicitly examine plant-based diets and their impact on chronic disease risk, published in English, and with clear methodologies.

We excluded studies focusing solely on animal models or in vitro experiments and studies without a clear definition of plant-based diets. We also excluded non-longitudinal studies (e.g., cross-sectional studies), case reports, editorials, commentaries, theses, and review articles. Studies with insufficient methodological clarity or unclear outcomes and those published without peer review were excluded as well.

Study Selection Process

The study selection process followed a structured approach to ensure objectivity and minimize bias, and it was done in three steps. During initial screening, titles and abstracts of all identified studies were screened for relevance based on the inclusion and exclusion criteria. Then, the full-text review focused on studies that passed the initial screening and were subjected to a full-text review to assess their eligibility. Finally, data from eligible studies were extracted using a standardized form, including study design, sample size, population characteristics, dietary interventions, outcomes, and key findings.

Two independent reviewers conducted the screening and data extraction processes. Discrepancies were resolved through discussion and consensus, with a third reviewer consulted.

Data Extraction and Management

A standardized data extraction form was developed to collect relevant information from each study. The following data were extracted: authors, year of publication, study design, sample size, type of plant-based diet (e.g., vegan, vegetarian, Mediterranean), duration of intervention, and dietary adherence measures. Outcomes, such as biomarkers (e.g., HbA1c, cholesterol levels) and disease incidence, were extracted along with other main findings of each study.

Quality Assessment

The methodological quality of the included studies was assessed using appropriate tools based on study design. The Cochrane Risk of Bias Tool [19] was used to evaluate randomization, blinding, allocation concealment, and reporting biases for randomized controlled trials (RCTs). The Newcastle-Ottawa Scale (NOS) [20] was used to assess selection, comparability, and outcome measurement for observational studies. For pilot and feasibility studies, the Mixed Methods Appraisal Tool (MMAT) [21] was used to evaluate methodological rigor. Studies were categorized as low, moderate, or high risk of bias based on their scores. Only studies with moderate to low risk of bias were included in the final synthesis.

Data Synthesis and Reporting

A narrative synthesis approach was employed to summarize the findings due to the heterogeneity of study designs, populations, and outcomes. The synthesis involved a thematic analysis, grouping studies by outcomes (e.g., diabetes prevention, cardiovascular health, weight management) and identifying common themes. The Preferred Reporting Items for Systematic Reviews and Meta-Analyses (PRISMA) standards were followed while reporting the results of the systematic review [22], and a thorough discussion of the findings' implications for public health policy and practice, as well as suggestions for further study, was conducted.

Results

**Figure 1:**
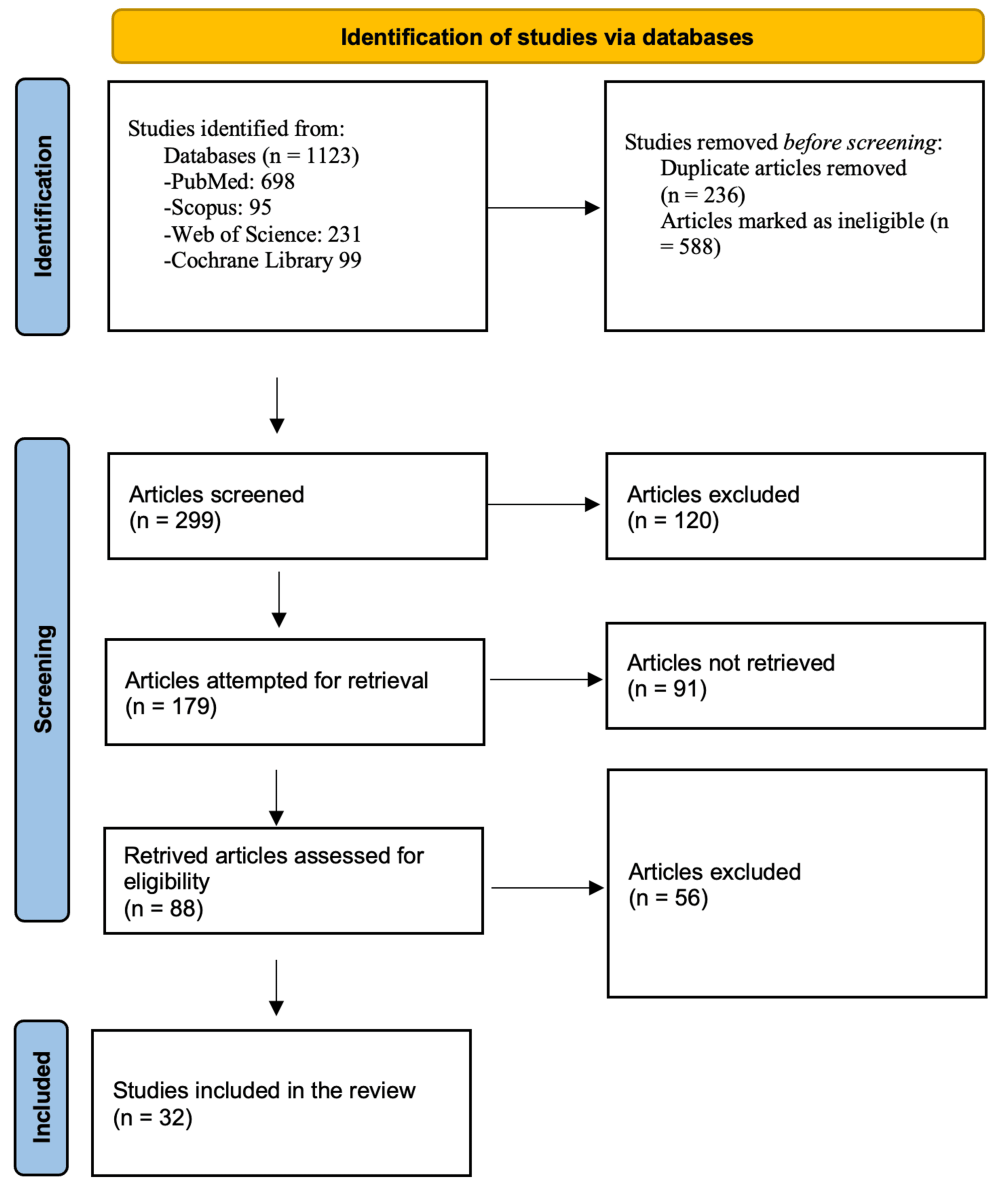
Selection process

The first search of the database turned up 1123 studies in total. A total of 299 titles and abstracts were evaluated for eligibility after duplicates were eliminated and the first elimination based on titles only. Following the additional removal of titles and abstracts that were deemed ineligible, 179 full-text articles were attempted to be retrieved, and 91 of them were unsuccessful. After retrieving 88 full-text publications and carefully evaluating them using the inclusion and exclusion criteria, 32 papers were found fully eligible and added to the systematic review (Figure 1).

Characteristics of the Included Studies

The 32 included studies explored the associations between plant-based diets and various health outcomes, including insulin resistance, type 2 diabetes, weight management, cardiovascular disease, metabolic syndrome, and other chronic conditions. The studies employ a variety of longitudinal designs, including observational studies (e.g., cohorts), RCTs, and pilot interventions, providing a comprehensive overview of the current evidence (Table 1). The sample size ranged from 13 to 16,781 participants, with most studies including hundreds of participants.

**Table 1:** Included studies *: year of publication BMI: body mass index; HDL: high-density lipoprotein; ALT: alanine aminotransferase; PB: plant-based; AHA: American Heart Association; LDL: low-density lipoprotein; WPFD: Whole Plant Food Density; MGUS: monoclonal gammopathy of undetermined significance; SMM: smoldering multiple myeloma; HOMA-IR: Homeostasis Model Assessment of Insulin Resistance; LFD: low-fat, high-fiber diet; iSAD: improved standard American diet; QoL: quality of life; hPDI: healthy plant-based diet index; MetS: metabolic syndrome; uPDI: unhealthy plant-based diet index; CKD: chronic kidney disease; TMAO: trimethylamine N-oxide; hsCRP: high-sensitivity C-reactive protein; sOB-R: soluble leptin receptor; DASH: Dietary Approaches to Stop Hypertension; PDI: plant-based diet index

No.	Title	Authors/year*	Study design	Sample	Main findings
1	Plant Versus Animal Based Diets and Insulin Resistance, Prediabetes and Type 2 Diabetes: The Rotterdam Study	Chen et al. [23] 2018	The study design is a prospective, population-based, multi-cohort observational study that used longitudinal analyses to examine the associations between a plant-based dietary index and insulin resistance, prediabetes, and type 2 diabetes	6798 participants	A higher adherence to a plant-based diet was associated with lower insulin resistance, lower risk of prediabetes, and lower risk of type 2 diabetes. The association between plant-based diet and lower insulin resistance and type 2 diabetes risk remained significant even after adjusting for BMI, but the association with prediabetes risk was no longer significant. The findings suggest that a more plant-based and less animal-based diet may lower the risk of insulin resistance, prediabetes, and type 2 diabetes
2	Adherence to a Plant-Based Diet and Consumption of Specific Plant Foods-Associations with 3-Year Weight-Loss Maintenance and Cardiometabolic Risk Factors: A Secondary Analysis of the PREVIEW Intervention Study	Zhu et al. [24] 2021	The study design was a 3-year, large-scale, 2×2 factorial randomized controlled trial conducted at 8 study centers across multiple countries	710 participants	Adherence to an overall plant-based diet was associated with less weight regain but not improvements in cardiometabolic risk factors. Consumption of specific plant foods like nuts, fruits, and vegetables was associated with improvements in weight management and cardiometabolic health. The associations with cardiometabolic risk factors were independent of weight changes
3	Coronary Risk Factors and Its Reduction by Plant-Based Diet with Emphasis on Diabetes: A Preliminary Report	Panigrahi [25] 2021	The study design is a prospective observational study with two parts: Part A to determine the degree of risk factors contributing to coronary artery disease and Part B to determine the effect of a plant-based diet on reversing type 2 diabetes	825 participants: 805 in Part A and 20 in Part B	Plant-based diet improved coronary risk factors, including a decrease in HbA1c, in patients with diabetes. The reduction in HbA1c was associated with a reduction in the number of antidiabetic medications in the compliant group
4	Implementation of a Whole Food Plant Based Diet in a Food as Prevention Program in a Resource Constrained Environment	Ganguli et al. [26] 2022	This study used a non-controlled, single-arm implementation design to evaluate the "Food as Prevention" program led by a single clinician. Participants self-selected to participate in the program and attended group sessions over 6-11 weeks	17 participants	Adoption of a whole food plant-based diet led to significant decreases in weight, total cholesterol, and triglycerides, as well as an increase in HDL cholesterol. The diet also led to borderline improvements in HbA1c and ALT. The study demonstrates that a whole food plant-based diet intervention run by a single clinician in a small group setting can produce meaningful improvements in multiple clinical parameters
5	Plant-Based, No-Added-Fat or American Heart Association Diets: Impact on Cardiovascular Risk in Obese Children with Hypercholesterolemia and Their Parents	Macknin et al. [27] 2015	A prospective, randomized, controlled trial with a 4-week duration was conducted in a large Midwestern hospital system's outpatient pediatric practices. 30 children (9-18 years old) and their parent pairs were randomly assigned to either a PB diet or the AHA diet and received weekly 2-hour nutrition education classes	60 participants (30 child-parent pairs)	Both the PB and AHA diets led to statistically significant beneficial changes in cardiovascular risk factors in children, with the PB diet showing more improvements. The PB diet also led to more beneficial changes in cardiovascular risk factors in adults compared to the AHA diet. The only significant change favoring the AHA diet over the PB diet was a slightly greater reduction in waist circumference in the children
6	Implementation of a Plant-Based, Nutrition Program in a Large Integrated Health Care System: Results of a Pilot Program	Rahman et al. [28] 2021	Retrospective, non-randomized, observational study with a matched comparison group	408 participants	Participation in the plant-based nutrition program was associated with improvements in health outcomes, including reductions in cholesterol levels, medication usage, office visits, and body weight. The study demonstrates that it is possible to implement a successful plant-based nutrition program in a large integrated healthcare system, despite the barriers that often prevent the use of plant-based diets in clinical practice
7	Nutrient-Dense, Plant-Rich Dietary Intervention Effective at Reducing Cardiovascular Disease Risk Factors for Worksites: A Pilot Study	Sutliffe et al. [29] 2016	The study design was a 6-week pre-post pilot intervention conducted at a single site (Northern Arizona University) without a control group	35 participants	The nutrient-dense, plant-rich dietary intervention resulted in significant improvements in weight, BMI, waist and hip measurements, HDL, LDL, and estimated average glucose. The worksite-based, nutrient-dense, plant-rich dietary intervention was an effective approach for reducing cardiovascular disease risk factors
8	Cardiometabolic Effects of Omnivorous vs Vegan Diets in Identical Twins: A Randomized Clinical Trial	Landry et al. [30] 2023	Randomized, controlled, crossover trial with identical twin pairs, 8-week intervention	44 participants	Participants on a healthy vegan diet had significantly lower LDL cholesterol levels compared to those on a healthy omnivorous diet. Participants on a healthy vegan diet had significantly lower fasting insulin levels compared to those on a healthy omnivorous diet. Participants on a healthy vegan diet experienced greater weight loss compared to those on a healthy omnivorous diet
9	Effects of 7 Days on an Ad Libitum Low-Fat Vegan Diet: The McDougall Program Cohort	McDougall et al. [31] 2014	Retrospective observational study	1615 participants	A low-fat, starch-based, vegan diet eaten ad libitum for 7 days resulted in significant favorable changes in biomarkers related to cardiovascular disease and metabolic disease risk, including weight loss, reduced cholesterol, blood pressure, and blood glucose. The diet led to a significant reduction in estimated 10-year cardiovascular disease risk, from over 7.5% to 5.5%. These improvements occurred even with many participants reducing or stopping their medications
10	Relapse Prevention by Plant-Based Diet Incorporated into Induction Therapy for Ulcerative Colitis: A Single-Group Trial	Chiba et al. [32] 2019	The study design was a single-group, non-randomized, non-controlled observational study that provided a plant-based diet to all participants during their hospitalization for ulcerative colitis induction therapy	92 participants	Relapse rates were much lower in patients with initial episodes of ulcerative colitis (14% at 1 year, 27% at 5 years) compared to those with relapses (36% at 1 year, 53% at 5 years). Adherence to the plant-based diet was significantly improved even at long-term follow-up (6 years 4 months). The study concluded that a plant-based diet is effective for preventing relapse of ulcerative colitis
11	Virtual Vegan Culinary Medicine Randomized Crossover Trial Improves Diet Quality in Patients at Risk for Heart Disease	Krenek et al. [33] 2023	Randomized, crossover, clinical trial	40 participants	Participation in a virtual vegan culinary medicine intervention increased diet quality, as measured by the WPFD index, in adults at risk for cardiovascular disease. All subcomponents of the WPFD index, including whole grains, legumes, whole fruits, vegetables, and nuts/seeds, significantly increased compared to the baseline. The WPFD index increased from 2.93 cup/oz-equivalents per 1000 kcal at baseline to 4.96 and 6.41 cup/oz-equivalents per 1000 kcal during the high and low extra virgin olive oil diet phases, respectively
12	The BROAD Study: A Randomised Controlled Trial Using a Whole Food Plant-Based Diet in the Community for Obesity, Ischaemic Heart Disease or Diabetes	Wright et al. [34] 2017	The study design was a randomized, controlled trial with a whole food plant-based diet as the intervention and normal care as the control. The primary endpoints were BMI and cholesterol, measured at 6 months and then extended to 12 months	Total: 65 participants. Control group: 32 participants. Intervention group: 33 participants	The WFPB diet led to a significantly greater reduction in BMI compared to normal care at 6 months. The WFPB diet led to a greater reduction in cholesterol compared to normal care, but this difference was not statistically significant unless dropouts were excluded. At 12 months, the WFPB diet group had a mean reduction of 4.2 BMI points and 0.55 mmol/L in total cholesterol
13	A Pilot Plant Based Dietary Intervention in MGUS and SMM Patients with Elevated BMI Is Feasible and Associated with Improvements in Metabolic and Microbiome Biomarkers of Progression	Shah et al. [35] 2022	A single-arm, non-randomized, non-controlled pilot study of a whole food plant-based diet intervention in patients with MGUS or SMM who have a BMI of 25 or greater	22 participants	The study met its primary feasibility endpoints, with a 7% median BMI reduction and 90% median adherence at 12 weeks. The whole food plant-based diet intervention was associated with improvements in metabolic markers, gut microbiome composition, and inflammatory biomarkers. The observed improvements in these biomarkers suggest potential mechanisms by which the dietary intervention could impact the progression of plasma cell disorders
14	Benefits of the Mediterranean Diet: Insights from the PREDIMED Study	Martínez-González et al. [36] 2015	The study design was a multicenter, randomized, primary prevention trial	7447 participants	The Mediterranean diet supplemented with extra-virgin olive oil or nuts resulted in a 30% lower risk of cardiovascular disease events compared to the control group. The Mediterranean diet supplemented with extra-virgin olive oil resulted in a 40% lower risk of developing diabetes, while the Mediterranean diet with nuts had a smaller, non-significant reduction in diabetes risk. The Mediterranean diet, with or without supplements, led to improvements in various cardiovascular disease risk factors such as blood pressure, insulin sensitivity, lipid profiles, inflammation, and atherosclerosis
15	A Plant-Based Dietary Intervention Improves Beta-Cell Function and Insulin Resistance in Overweight Adults: A 16-Week Randomized Clinical Trial	Kahleova et al. [37] 2018	Randomized, single-center, parallel-group study with an intervention group following a low-fat vegan diet and a control group making no diet changes, with assessments at baseline and 16 weeks	75 participants: 38 in the intervention group and 37 in the control group	The dietary intervention group showed a marked increase in meal-stimulated insulin secretion compared to the control group. The dietary intervention group had a significant decrease in insulin resistance as measured by the HOMA-IR index. The improvements in insulin resistance were related to reductions in visceral fat, independent of changes in BMI
16	Prevention of Diabetes With Mediterranean Diets: A Subgroup Analysis of a Randomized Trial	Salas-Salvadó et al. [38] 2014	The study design was a parallel-group, randomized, primary cardiovascular prevention trial. It was a multi-site study conducted in Spain. Participants were randomly assigned in a 1:1:1 ratio to one of three nutrition interventions: Mediterranean diet supplemented with extra-virgin olive oil, Mediterranean diet supplemented with mixed nuts, or a control diet with advice to reduce the intake of all types of fat. The study was not blinded, but the investigators assessing outcomes were blinded to the intervention assignment	3541 participants	Participants assigned to Mediterranean diet groups, supplemented with either extra-virgin olive oil or mixed nuts, had a lower incidence of diabetes compared to the control group that received advice to reduce fat intake after 4 years of follow-up
17	An Apple a Day Keeps the Doctor Away: The Effect of a Low-Fat, High-Fiber Diet on Quality of Life, Inflammation, and Dysbiosis in Patients with Ulcerative Colitis	Gold and Cohen-Mekelburg [39] 2022	Randomized, crossover study	38 participants were enrolled in the study, and 17 participants (44.7%) were included in the final analysis	Both the LFD and the iSAD led to improvements in QoL and clinical symptoms for the patients who completed the study. The LFD was associated with a higher QoL than the iSAD. The LFD was associated with changes in the gut microbiome, including an increase in *Faecalibacterium prausnitzii* and *Prevotella* species, compared to the iSAD and baseline
18	Evaluation of an Eight-Week Whole-Food Plant-Based Lifestyle Modification Program	Campbell et al. [40] 2017	The study design was a non-randomized, non-controlled group program or intervention, with participants enrolled in small cohorts and the program repeated 7 times with new cohorts	79 participants	The 8-week whole-food plant-based intervention resulted in significant weight loss, with participants with higher BMI at baseline losing a greater percentage of their body weight. The intervention also resulted in significant reductions in blood pressure and cholesterol levels. Over a quarter of participants were able to decrease or discontinue at least one chronic medication
19	A Multicenter Randomized Controlled Trial of a Plant-Based Nutrition Program to Reduce Body Weight and Cardiovascular Risk in the Corporate Setting: The GEICO Study	Mishra et al. [41] 2013	The study design was a multicenter, randomized, controlled, parallel-group trial of an 18-week dietary intervention using a low-fat plant-based diet in a corporate setting	142 at intervention sites and 149 at control sites	The plant-based diet intervention led to significantly greater weight loss compared to the control group. The plant-based diet intervention led to significantly greater reductions in total and LDL cholesterol compared to the control group. For participants with diabetes, the plant-based diet intervention led to a significantly greater reduction in HbA1C (a measure of glycemic control) compared to the control group
20	Effects of an Ad Libitum Consumed Low-Fat Plant-Based Diet Supplemented with Plant-Based Meal Replacements on Body Composition Indices	Jakše et al. [42] 2017	Non-randomized, controlled, interventional trial	Total: 325. Intervention group: 241. Control group: 84	The intervention group experienced significant reductions in body fat percentage (4.3% points), visceral fat (1.6 units), and body weight (5.6 kg) compared to the control group. The intervention group experienced a negligible reduction in muscle mass, with a relative increase in muscle mass percentage of 4.2% points. 60% of the intervention group continued to lose weight after the 10-week program, with an average additional weight loss of 3.3 kg
21	Diabetes Reversal by Plant-Based Diet	Chowdhury [43] 2017	The study design was a 3-day, single-arm, uncontrolled clinical trial on 55 diabetes patients, where participants discontinued their medications and followed a plant-based diet. The study collected fasting and post-prandial blood glucose readings, as well as participant weights, and included patients with varying diabetes history, age, type of diabetes, and insulin dependency	55 participants	84% of patients achieved controlled blood glucose levels without any medications or insulin, and 16% achieved partially controlled levels with significantly reduced insulin requirements, all within 3 days of following the prescribed plant-based diet. 100% of type 2 diabetes patients achieved healthy blood glucose levels, while 57% of type 1 diabetes patients achieved controlled levels without any medications, and 43% achieved healthy levels with reduced insulin. 59% of insulin-dependent patients were able to completely eliminate their insulin requirements, and 41% were able to reduce their insulin by at least 50%
22	A Plant-Based Diet in Overweight Individuals in a 16-Week Randomized Clinical Trial: Metabolic Benefits of Plant Protein	Kahleova et al. [44] 2018	The study design was a 16-week randomized, parallel-group clinical trial comparing a plant-based diet to a control diet	Total: 75. Plant-based diet: 38. Control diet: 37	The plant-based vegan diet led to significantly greater reductions in body weight, fat mass, and insulin resistance compared to the control diet. The vegan group showed significant reductions in body weight (-6.5 kg), fat mass (-4.3 kg), and insulin resistance (HOMA-IR decreased by 1.0). The decrease in fat mass was associated with an increased intake of plant protein and a decreased intake of animal protein
23	The Association Between Plant-Based Diet Indices and Obesity and Metabolic Diseases in Chinese Adults: Longitudinal Analyses From the China Health and Nutrition Survey	Chen et al. [45] 2022	The study design is a prospective, population-based, longitudinal observational study using data from the China Health and Nutrition Survey from 2004 to 2015. It had a retrospective cohort design, where participants were followed over time and classified into different samples based on their baseline health status (overweight/obesity, hypertension, or type 2 diabetes)	16,781 participants	Higher adherence to plant-based diet indices (PDI and hPDI) was associated with reduced risk of obesity, hypertension, and type 2 diabetes in Chinese adults, especially those under 55 years old. The association between plant-based diet indices and reduced disease risk was stronger in younger adults (<55 years) compared to older adults. The association between plant-based diet indices and reduced obesity risk was stronger in women, while the association with reduced type 2 diabetes risk was seen in both men and women
24	The Association between Plant-Based Diet Indices and Metabolic Syndrome in Chinese Adults: Longitudinal Analyses from the China Health and Nutrition Survey	Huo et al. [46] 2023	Longitudinal observational cohort study	10,013 participants	A higher hPDI was associated with a lower risk of developing MetS and abdominal obesity. No significant association was found between the uPDI and MetS, but a higher uPDI was associated with a higher risk of abdominal obesity. Baseline BMI mediated a significant portion of the association between hPDI and the risk of MetS and abdominal obesity
25	Replacing Animal-Based Proteins with Plant-Based Proteins Changes the Composition of a Whole Nordic Diet—A Randomised Clinical Trial in Healthy Finnish Adults	Päivärinta et al. [47] 2020	Randomized, parallel-group clinical trial	145 participants	Replacing animal proteins with plant-based proteins in the diet decreased overall protein intake but maintained recommended levels. Increasing plant proteins in the diet increases fiber intake and improves the fatty acid composition by reducing saturated fats and increasing polyunsaturated fats. The changes in dietary fatty acid composition led to a significant improvement in blood lipoprotein profile
26	Relapse Prevention in Ulcerative Colitis by Plant-Based Diet Through Educational Hospitalization: A Single-Group Trial	Chiba et al. [48] 2018	Non-randomized, non-controlled, single-group observational study	60 participants	The relapse rates in ulcerative colitis patients were very low after a plant-based diet intervention, with only 19% relapsing over 5 years of follow-up. Most patients experienced immediate improvements in symptoms like decreased bloody stool during the plant-based diet hospitalization. Participants were able to maintain the plant-based diet long-term, as indicated by higher PBD scores after the hospitalization
27	The Effect of a Diet Containing 70% Protein from Plants on Mineral Metabolism and Musculoskeletal Health in Chronic Kidney Disease	Moorthi et al. [49] 2014	A single-arm, non-controlled, 4-week intervention study where 13 participants with CKD 3-4 received a diet containing 70% protein from plants	13 participants	A 70% plant protein diet significantly decreased urine phosphorus excretion in CKD patients. The 70% plant protein diet did not significantly affect serum levels of FGF23, phosphorus, or PTH in CKD patients. The 70% plant protein diet decreased urine sodium and titratable acid in CKD patients
28	A Randomized Crossover Trial on the Effect of Plant-Based Compared with Animal-Based Meat on Trimethylamine-N-Oxide and Cardiovascular Disease Risk Factors in Generally Healthy Adults: Study With Appetizing Plantfood-Meat Eating Alternative Trial (SWAP-MEAT)	Crimarco et al. [50] 2020	The study design was a single-site, randomized, controlled crossover trial with no washout period	36 participants	Consuming plant-based meat alternatives resulted in lower TMAO levels compared to consuming animal meat, but the order in which the diets were consumed affected the results. Consuming plant-based meat alternatives resulted in lower LDL cholesterol levels and lower body weight compared to consuming animal meat
29	Change in Plant-Based Diet Quality Is Associated with Changes in Plasma Adiposity-Associated Biomarker Concentrations in Women	Baden et al. [51] 2019	The study design was a longitudinal, observational cohort study that followed a sample of women from the Nurses' Health Study II over an average of 13 years, collecting dietary data and blood samples at two time points	831 women	A healthful plant-based diet was associated with lower concentrations of leptin, insulin, and hsCRP and higher concentrations of adiponectin and sOB-R, even after adjusting for BMI. An increase in healthful plant-based diet quality over time was inversely associated with changes in leptin and hsCRP, whereas an increase in unhealthful plant-based diet quality was positively associated with changes in leptin, hsCRP, and IL-6, even after adjusting for weight change. The results provide evidence for the anti-inflammatory and metabolic benefits of a healthful plant-based diet
30	The Acute Effects of a DASH diet and Whole Food, Plant-Based diet on Insulin Requirements and Related Cardiometabolic Markers in Individuals with Insulin-Treated Type 2 Diabetes	Campbell et al. [52] 2023	Non-randomized crossover trial	15 participants	Adopting a DASH or WFPB diet led to significant reductions in daily insulin usage among individuals with insulin-treated type 2 diabetes. The WFPB diet led to improvements in insulin resistance and insulin sensitivity, which then regressed during the subsequent DASH 2 diet. The WFPB diet led to improvements in various cardiometabolic markers, which then worsened during the subsequent DASH 2 diet
31	Plant‐Based Diet Index and Erectile Dysfunction in the Health Professionals Follow‐Up Study	Yang et al. [53] 2022	The study design is an observational, prospective cohort study that is part of the larger Health Professionals Follow-Up Study. It is a longitudinal study evaluating the association between PDI score and the incidence of erectile dysfunction	21,942 men aged 40-75 years	In men between the ages of 60 and less than 70, incident erectile dysfunction was negatively correlated with hPDI. In that age group, the risk of erectile dysfunction was 18% lower for those in the highest quintile of hPDI than for those in the lowest quintile (HR=0.82; 95% CI=0.73-0.91; P-trend<0.001). On the other hand, among males under 60, uPDI was positively correlated with erectile dysfunction (HR=1.27; 95% CI=1.01-1.60; P-trend=0.02)
32	Chronic Musculoskeletal Pain and Function Improve with a Plant-Based Diet	Towery et al. [54] 2018	Longitudinal quasi-experimental cohort study	14 participants completed the study	The plant-based diet intervention resulted in decreased chronic pain and improved QoL for the participants. The majority of participants (10 out of 14) were able to adhere to the plant-based diet at a high rate of 89%. Consumption of the plant-based diet produced positive improvements in chronic pain and function

The key findings showed that plant-based diets were consistently associated with improved metabolic health, weight management, cardiovascular risk reduction, and better management of chronic conditions like diabetes and ulcerative colitis.

Insulin Resistance, Prediabetes, and Type 2 Diabetes

Included studies consistently demonstrate that higher adherence to plant-based diets is associated with lower insulin resistance, reduced risk of prediabetes, and a lower incidence of type 2 diabetes. Chen et al. [23] found that a higher plant-based dietary index was associated with lower insulin resistance and a reduced risk of type 2 diabetes, even after adjusting for body mass index (BMI). Similarly, Kahleova et al. [37] reported significant improvements in insulin secretion and insulin resistance in participants following a low-fat vegan diet compared to controls. Notably, Hanick et al. [55] observed dramatic improvements in blood glucose control in diabetes patients adopting a plant-based diet, eliminating insulin requirements. Similar findings were also reported by Panigrahi [25] and Ganguli et al. [26].

Weight Management

Plant-based diets appear to be effective in promoting weight loss and maintaining weight loss over time. Macknin et al. [27] found that a plant-based diet led to greater weight loss in obese hypercholesterolemic children compared to the American Heart Association diet. Additionally, the BROAD study [34] showed that a whole food plant-based diet resulted in significant reductions in BMI and cholesterol levels over 12 months. Jakše et al. [42] reported significant reductions in body fat percentage and visceral fat in participants following a low-fat plant-based diet supplemented with meal replacements.

Cardiovascular Disease

The evidence suggests that plant-based diets can improve cardiovascular health by reducing cholesterol levels, lowering blood pressure, and decreasing the risk of cardiovascular events [30,31,40,41]. Landry et al. [30] found that a healthy vegan diet led to significant reductions in low-density lipoprotein (LDL) cholesterol and fasting insulin levels compared to an omnivorous diet. Similarly, Martínez-González et al. [36] and Salas-Salvadó et al. [38] demonstrated that a Mediterranean diet supplemented with extra-virgin olive oil or nuts reduced the risk of cardiovascular disease events by 30%. McDougall et al. [31] and Sutliffe et al. [29] also showed significant improvements in cardiovascular risk factors, including weight loss and reduced cholesterol levels, even with ad libitum food intake. Integration of virtual vegan culinary improves diet quality in patients at risk for heart disease due to increased diet quality [33].

Metabolic Syndrome and Obesity

Higher adherence to plant-based diets is associated with a reduced risk of metabolic syndrome and obesity. Huo et al. [46] found that a higher healthy plant-based diet index (hPDI) was associated with a lower risk of developing metabolic syndrome, particularly in younger adults. Similarly, Chen et al. [45] and Kahleova et al. [44] reported that higher adherence to plant-based diet indices was associated with reduced risk of obesity, hypertension, and type 2 diabetes in Chinese adults, with stronger associations in younger individuals and women. Rahman et al. [28] reported that plant-based nutrition led to reductions in cholesterol levels, medication usage, office visits, and body weight, which can improve health outcomes and prevent metabolic syndrome and obesity. This is supported also by significant improvements in weight, BMI, waist and hip measurements, high-density lipoprotein (HDL), LDL, and estimated average glucose reported by other studies [29,30].

Chronic Inflammatory and Autoimmune Conditions

Plant-based diets may also play a role in managing chronic inflammatory and autoimmune conditions. Two studies [32,48] observed lower relapse rates in ulcerative colitis patients who adhered to a plant-based diet, with improvements in symptoms and quality of life. Gold and Cohen-Mekelburg [39] found that a low-fat, high-fiber diet improved the quality of life and clinical symptoms in patients with ulcerative colitis, with beneficial changes in the gut microbiome, similar to the findings of Shah et al. [35].

Muscle Health and Chronic Pain

Contrary to concerns about muscle loss, some studies suggest that plant-based diets can improve muscle health and reduce chronic pain. Jakše et al. [42] reported a relative increase in muscle mass percentage in participants following a plant-based diet, while Towery et al. [54] found that a plant-based diet improved chronic pain and quality of life in participants with musculoskeletal pain.

Mechanisms and Considerations

The beneficial effects of plant-based diets are likely attributed to their high content of fiber, antioxidants, and phytochemicals, which may improve insulin sensitivity, reduce inflammation, and enhance gut microbiome health [39,50,51,53]. Additionally, the lower intake of saturated fats and cholesterol from animal products may contribute to improvements in cardiovascular health [47,49,52]. However, it is important to consider potential limitations, such as the challenges of adherence to plant-based diets and the need for careful nutrient planning to ensure adequate intake of essential nutrients.

This review identified some inconsistencies, gaps, and patterns in the literature regarding the role of plant-based diets in the prevention of chronic diseases (Table 2). 

**Table 2:** Patterns, inconsistencies, and gaps identified LDL: low-density lipoprotein; RCTs: randomized controlled trials

Category	Description
Patterns	
1. Metabolic health	Plant-based diets consistently improve insulin sensitivity and glycemic control and reduce the risk of type 2 diabetes [23,44]
2. Weight management	Plant-based diets are effective for weight loss and weight maintenance, with reductions in body fat and visceral fat [34,42]
3. Cardiovascular health	Plant-based diets lower LDL cholesterol, blood pressure, and overall cardiovascular risk [30,31]
4. Gut microbiome	Plant-based diets improve gut microbiome composition and reduce systemic inflammation [35,39]
Inconsistencies	
1. Diet definitions	Variability in defining plant-based diets (e.g., whole food vs. processed plant-based diets) leads to mixed outcomes [46,50]
2. Specific outcomes	Some studies report no improvement in cardiometabolic risk factors despite weight loss [24]
3. Population heterogeneity	Effects of plant-based diets vary across populations (e.g., healthy adults vs. chronic disease patients), with some groups showing greater benefits [27,55]
Gaps	
1. Long-term studies	Lack of long-term studies assessing the sustainability and durability of plant-based diets [32]
2. Mechanistic insights	Limited exploration of how specific components of plant-based diets (e.g., fiber, polyphenols) influence metabolic pathways and gut microbiota
3. Diverse populations	Underrepresentation of diverse populations and cultural contexts, particularly in low- and middle-income countries [45,46]
4. Intervention studies	Few RCTs with rigorous methodologies; many studies lack control groups [26,35]
5. Personalized nutrition	Limited research on personalized plant-based dietary interventions based on genetic, metabolic, and microbiome profiles

Risk of Bias Assessment

Most RCTs were assessed as having a low risk of bias, with some studies showing moderate risk due to issues with blinding or allocation concealment. Observational studies generally had a low to moderate risk of bias, with some studies scoring lower due to issues with comparability or outcome measurement. Pilot studies were mostly rated as having a low risk of bias, with a few exceptions where recruitment or data collection methods introduced moderate risk. Studies that indicated high risk were excluded and were not added to this review. The risk of bias assessment results of included studies are shown in Table 3 and show the different tools used depending on the study design.

**Table 3:** Risk of bias assessment results NOS: Newcastle-Ottawa Scale; MMAT: Mixed Methods Appraisal Tool

No.	Title	Authors/year	Study design	Risk of bias assessment	Risk of bias level
1	Plant Versus Animal Based Diets and Insulin Resistance, Prediabetes and Type 2 Diabetes: The Rotterdam Study	Chen et al. [23] 2018	Prospective, population-based, multi-cohort observational study	Low risk of bias (NOS score: 8/9)	Low
2	Adherence to a Plant-Based Diet and Consumption of Specific Plant Foods-Associations with 3-Year Weight-Loss Maintenance and Cardiometabolic Risk Factors: A Secondary Analysis of the PREVIEW Intervention Study	Zhu et al. [24] 2021	3-year, large-scale, 2×2 factorial randomized, controlled trial	Moderate risk of bias (Cochrane Tool: some concerns in blinding and allocation concealment)	Moderate
3	Coronary Risk Factors and Its Reduction by Plant-Based Diet with Emphasis on Diabetes: A Preliminary Report	Panigrahi [25] 2021	Prospective observational study	Moderate risk of bias (NOS score: 7/9)	Moderate
4	Implementation of a Whole Food Plant Based Diet in a Food as Prevention Program in a Resource Constrained Environment	Ganguli et al. [26] 2022	Non-controlled, single-arm implementation design	Moderate risk of bias (MMAT score: 3/5)	Moderate
5	Plant-Based, No-Added-Fat or American Heart Association Diets: Impact on Cardiovascular Risk in Obese Children with Hypercholesterolemia and Their Parents	Macknin et al. [27] 2015	Prospective, randomized, controlled trial	Low risk of bias (Cochrane Tool: low risk in all domains)	Low
6	Implementation of a Plant-Based, Nutrition Program in a Large Integrated Health Care System: Results of a Pilot Program	Rahman et al. [28] 2021	Retrospective, non-randomized, observational study	Moderate risk of bias (NOS score: 6/9)	Moderate
7	Nutrient-Dense, Plant-Rich Dietary Intervention Effective at Reducing Cardiovascular Disease Risk Factors for Worksites: A Pilot Study	Sutliffe et al. [29] 2016	6-week pre-post pilot intervention	Moderate risk of bias (MMAT score: 3/5)	Moderate
8	Cardiometabolic Effects of Omnivorous vs Vegan Diets in Identical Twins: A Randomized Clinical Trial	Landry et al. [30] 2023	Randomized, controlled, crossover trial	Low risk of bias (Cochrane Tool: low risk in all domains)	Low
9	Effects of 7 Days on an Ad Libitum Low-Fat Vegan Diet: The McDougall Program Cohort	McDougall et al. [31] 2014	Retrospective observational study	Moderate risk of bias (NOS score: 6/9)	Moderate
10	Relapse Prevention by Plant-Based Diet Incorporated into Induction Therapy for Ulcerative Colitis: A Single-Group Trial	Chiba et al. [32] 2019	Single-group, non-randomized, non-controlled observational study	Moderate risk of bias (MMAT score: 3/5)	Moderate
11	Virtual Vegan Culinary Medicine Randomized Crossover Trial Improves Diet Quality in Patients at Risk for Heart Disease	Krenek et al. [33] 2023	Randomized, crossover, clinical trial	Low risk of bias (Cochrane Tool: low risk in all domains)	Low
12	The BROAD Study: A Randomised Controlled Trial Using a Whole Food Plant-Based Diet in the Community for Obesity, Ischaemic Heart Disease or Diabetes	Wright et al. [34] 2017	Randomized, controlled trial	Low risk of bias (Cochrane Tool: low risk in all domains)	Low
13	A Pilot Plant Based Dietary Intervention in MGUS and SMM Patients with Elevated BMI Is Feasible and Associated with Improvements in Metabolic and Microbiome Biomarkers of Progression	Shah et al. [35] 2022	Single-arm, non-randomized, non-controlled pilot study	Moderate risk of bias (MMAT score: 3/5)	Moderate
14	Benefits of the Mediterranean Diet: Insights from the PREDIMED Study	Martínez-González et al. [36] 2015	Multicenter, randomized, primary prevention trial	Low risk of bias (Cochrane Tool: low risk in all domains)	Low
15	A Plant-Based Dietary Intervention Improves Beta-Cell Function and Insulin Resistance in Overweight Adults: A 16-Week Randomized Clinical Trial	Kahleova et al. [37] 2018	Randomized, single-center, parallel-group study	Low risk of bias (Cochrane Tool: low risk in all domains)	Low
16	Prevention of Diabetes With Mediterranean Diets: A Subgroup Analysis of a Randomized Trial	Salas-Salvadó et al. [38] 2014	Parallel-group, randomized, primary cardiovascular prevention trial	Low risk of bias (Cochrane Tool: low risk in all domains)	Low
17	An Apple a Day Keeps the Doctor Away: The Effect of a Low-Fat, High-Fiber Diet on Quality of Life, Inflammation, and Dysbiosis in Patients with Ulcerative Colitis	Gold and Cohen-Mekelburg [39] 2022	Randomized, crossover study	Low risk of bias (Cochrane Tool: low risk in all domains)	Low
18	Evaluation of an Eight-Week Whole-Food Plant-Based Lifestyle Modification Program	Campbell et al. [40] 2017	Non-randomized, non-controlled group program	Moderate risk of bias (MMAT score: 3/5)	Moderate
19	A Multicenter Randomized Controlled Trial of a Plant-Based Nutrition Program to Reduce Body Weight and Cardiovascular Risk in the Corporate Setting: The GEICO Study	Mishra et al. [41] 2013	Multicenter, randomized, controlled, parallel-group trial	Low risk of bias (Cochrane Tool: low risk in all domains)	Low
20	Effects of an Ad Libitum Consumed Low-Fat Plant-Based Diet Supplemented with Plant-Based Meal Replacements on Body Composition Indices	Jakše et al. [42] 2017	Non-randomized, controlled, interventional trial	Moderate risk of bias (NOS score: 6/9)	Moderate
21	A Plant-Based Diet in Overweight Individuals in a 16-Week Randomized Clinical Trial: Metabolic Benefits of Plant Protein	Kahleova et al. [44] 2018	16-week randomized, parallel-group clinical trial	Low risk of bias (Cochrane Tool: low risk in all domains)	Low
22	The Association Between Plant-Based Diet Indices and Obesity and Metabolic Diseases in Chinese Adults: Longitudinal Analyses From the China Health and Nutrition Survey	Chen et al. [45] 2022	Prospective, population-based, longitudinal observational study	Low risk of bias (NOS score: 8/9)	Low
23	The Association between Plant-Based Diet Indices and Metabolic Syndrome in Chinese Adults: Longitudinal Analyses from the China Health and Nutrition Survey	Huo et al. [46] 2023	Longitudinal observational cohort study	Low risk of bias (NOS score: 8/9)	Low
24	Replacing Animal-Based Proteins with Plant-Based Proteins Changes the Composition of a Whole Nordic Diet—A Randomised Clinical Trial in Healthy Finnish Adults	Päivärinta et al. [47] 2020	Randomized, parallel-group clinical trial	Low risk of bias (Cochrane Tool: low risk in all domains)	Low
25	Relapse Prevention in Ulcerative Colitis by Plant-Based Diet Through Educational Hospitalization: A Single-Group Trial	Chiba et al. [48] 2018	Non-randomized, non-controlled, single-group observational study	Moderate risk of bias (MMAT score: 3/5)	Moderate
26	The Effect of a Diet Containing 70% Protein from Plants on Mineral Metabolism and Musculoskeletal Health in Chronic Kidney Disease	Moorthi et al. [49] 2014	Single-arm, non-controlled, 4-week intervention study	Moderate risk of bias (MMAT score: 3/5)	Moderate
27	A Randomized Crossover Trial on the Effect of Plant-Based Compared with Animal-Based Meat on Trimethylamine-N-Oxide and Cardiovascular Disease Risk Factors in Generally Healthy Adults: Study With Appetizing Plantfood-Meat Eating Alternative Trial (SWAP-MEAT)	Crimarco et al. [50] 2020	Randomized, controlled crossover trial	Low risk of bias (Cochrane Tool: low risk in all domains)	Low
28	Change in Plant-Based Diet Quality Is Associated with Changes in Plasma Adiposity-Associated Biomarker Concentrations in Women	Baden et al. [51] 2019	Longitudinal, observational cohort study	Low risk of bias (NOS score: 8/9)	Low
29	The Acute Effects of a DASH diet and Whole Food, Plant-Based diet on Insulin Requirements and Related Cardiometabolic Markers in Individuals with Insulin-Treated Type 2 Diabetes	Campbell et al. [52] 2023	Non-randomized crossover trial	Moderate risk of bias (MMAT score: 3/5)	Moderate
30	Plant‐Based Diet Index and Erectile Dysfunction in the Health Professionals Follow‐Up Study	Yang et al. [53] 2022	Observational, prospective cohort study	Low risk of bias (NOS score: 8/9)	Low
31	Chronic Musculoskeletal Pain and Function Improve with a Plant-Based Diet	Towery et al. [54] 2018	Longitudinal quasi-experimental cohort study	Moderate risk of bias (MMAT score: 3/5)	Moderate
32	A Whole-Food, Plant-Based Intensive Lifestyle Intervention Improves Glycaemic Control and Reduces Medications in Individuals with Type 2 Diabetes: A Randomised Controlled Trial	Hanick et al. [55] 2025	A randomized controlled trial compared 169 adults aged 18-75 with type 2 diabetes to an intensive whole-food, plant-based intervention for 24 weeks	Low risk of bias (Cochrane Tool: low risk in all domains)	Low

An RCT was classified as having a low risk of bias if it demonstrates low risk across all domains tested by the Cochrane Risk of Bias Tool, indicating a robust methodology with minimal potential for bias. If a study has some concerns in one or more domains, it was classified as having a moderate risk of bias, suggesting that while the study is generally well-conducted, there are areas where bias could influence the results. A study was classified as having a high risk of bias if it has a high risk in at least one domain, indicating significant methodological flaws that could substantially affect the validity of the findings.

Using the NOS, the observational studies were scored on a star system, with a maximum of 9 stars. A study scoring 7-9 stars was considered to have a low risk of bias, indicating high methodological quality with strong selection, comparability, and outcome assessment. Studies scoring 4-6 stars were classified as having a moderate risk of bias, suggesting some limitations in study design or execution that could introduce bias, while studies scoring 0-3 stars were considered to have a high risk of bias, indicating significant methodological weaknesses that likely compromise the reliability of the results.

The MMAT was used to assess studies based on five criteria, with each criterion scored as "yes" (1 point), "no" (0 points), or "can't tell" (0 points). A study scoring 4-5 points was classified as having a low risk of bias, indicating strong methodological rigor and minimal potential for bias. Studies scoring 2-3 points were considered to have a moderate risk of bias, suggesting some methodological limitations that could affect the study's validity. Studies scoring 0-1 point were classified as having a high risk of bias, indicating significant methodological flaws that likely undermine the credibility of the findings.

Discussion

This systematic review on plant-based diets highlights their significant role in preventive medicine, supported by a wealth of evidence from various studies. The included longitudinal studies reported on mechanisms, benefits, and challenges associated with plant-based diets, enriching the currently available knowledge.

Plant-based diets are rich in fiber, which plays a crucial role in gut health by promoting the growth of beneficial microbiota. Studies have shown that a high-fiber diet can reduce systemic inflammation, a key factor in chronic diseases. Additionally, phytochemicals, abundant in plant foods, exhibit antioxidant and anti-inflammatory properties that contribute to disease prevention [8,15,16]. These compounds, such as flavonoids and carotenoids, have been linked to reduced risk of cardiovascular diseases and certain cancers [43,56]. The review supports the role of plant-based diets in reducing inflammation and improving gut microbiome health, which are pivotal in chronic disease prevention. A previous systematic review suggests that plant-based diets enhance gut microbiota diversity, potentially mitigating inflammation and metabolic disorders [57].

The high fiber and water content in plant-based diets enhance satiety, leading to reduced calorie intake and aiding in weight management [58]. This is supported by studies showing lower BMI and reduced obesity rates among plant-based diet adherents [59]. Aligning with our findings, beyond cholesterol reduction, plant-based diets improve blood pressure and endothelial function, further benefiting cardiovascular health. These effects are attributed to the diets' low saturated fat and high potassium content [5]. A previous systematic review found that adherence to a Dietary Approaches to Stop Hypertension (DASH)-style diet reduces cardiovascular disease risk by 20%, 21%, 19%, and 29%, with a linear, inverse relationship between diet adherence and overall cardiovascular disease risk [60]. Similarly, we found that the DASH diet led to improvements in various cardiometabolic markers, as well as daily insulin usage among individuals with insulin-treated type 2 diabetes.

Furthermore, our review showed that a Mediterranean diet that is rich in plant-based nutrients also helps improve diabetes. Supporting this, a previous systematic review by Abbate et al. [61] showed that a Mediterranean diet without exercise was effective in reducing blood pressure, cardiovascular events, and risk of type 2 diabetes, while a low-fat diet was only effective when combined with exercise and weight loss. Plant-based diets, particularly those with a low glycemic index, help stabilize blood sugar levels, making them beneficial for diabetes management. Our findings have demonstrated improved glycemic control and reduced insulin resistance in diabetics following such diets, similar to previous systematic reviews [60,61]. The high fiber content also slows glucose absorption, preventing spikes in blood sugar.

The systematic review's findings align with previous studies indicating that plant-based diets are associated with lower insulin resistance, reduced diabetes risk, weight management, and improved cardiovascular health. The literature highlights the benefits of plant-based diets in managing chronic diseases, consistent with the current review's outcomes. However, discrepancies exist, such as studies suggesting that plant-based diets high in refined carbohydrates may not offer the same benefits, emphasizing the importance of diet quality. However, not all plant-based diets are equal; the quality and composition significantly influence health outcomes. Diets rich in whole grains, fruits, and vegetables are more beneficial than those high in processed foods. This underscores the need for dietary guidelines to emphasize whole, unprocessed plant foods. Moreover, different plant-based dietary patterns, such as vegan, vegetarian, and flexitarian, have varying health impacts. Vegan diets, excluding all animal products, show the most significant benefits in reducing chronic disease risk [62]. Flexitarian diets, which include occasional animal products, offer flexibility and may be more sustainable for some individuals [63]. These underline that there is a need for education targeting individuals with chronic conditions and the general public about diet and dietary habit change to improve health outcomes.

Promoting plant-based diets in public health policies can significantly reduce the burden of chronic diseases. Clinicians, along with nutritionists, can play a pivotal role by providing evidence-based dietary recommendations and addressing potential barriers. Integrating plant-based diets into healthcare practices may require education and training for healthcare providers to ensure effective implementation. Beyond health benefits, plant-based diets offer environmental advantages, such as reduced greenhouse gas emissions and resource conservation. A shift to plant-based diets might result in significant health co-benefits while reducing diet-related land usage by 76%, diet-related greenhouse gas emissions by 49%, eutrophication by 49%, and green and blue water consumption by 21% and 14%, respectively [64]. Economically, their cost-effectiveness in preventing chronic diseases could alleviate healthcare burdens. 

The integration of virtual plant-based diet cooking programs has the potential to significantly improve the awareness and adoption of plant-based diets, leading to a reduction in chronic disease risk. This is supported by one included study showing that virtual vegan culinary medicine improves diet quality in patients at risk for heart disease. By providing accessible, practical, and personalized education, these programs can empower individuals to make sustainable dietary changes that benefit their health, the environment, and society as a whole. However, addressing barriers such as digital access and long-term adherence will be critical to maximizing their impact.

Despite their benefits, plant-based diets may pose challenges. Nutrient deficiencies, such as vitamin B12, iron, and calcium, are potential risks, particularly in strictly vegan diets [65]. However, these can be mitigated through fortified foods and supplements. Cultural acceptability is another challenge, as traditional diets vary globally. Studies exploring cultural adaptation of plant-based diets should be conducted to inform tailored approaches to enhance the acceptance and adoption of nutrient-rich plant-based diets.

The systematic review highlights consistent patterns supporting the role of plant-based diets in improving metabolic health, weight management, and cardiovascular risk. However, inconsistencies in diet definitions, study designs, and population characteristics underscore the need for standardized methodologies and tailored interventions. Critical gaps, such as the lack of long-term studies, mechanistic insights, and diverse population representation, provide opportunities for future research. Addressing these gaps will enhance our understanding of plant-based diets and their potential to transform preventive medicine.

Promoting plant-based diets through public health policy changes can improve population health, reduce chronic disease burden, and support environmental sustainability. Key interventions include updating national dietary guidelines, integrating plant-based nutrition into healthcare training and school curriculums, and launching public awareness campaigns. Subsidies and taxes can make plant-based foods more affordable and tax unhealthy processed meats and ultra-processed foods, and ensuring plant-based options in public institutions, regulating food labeling, and encouraging urban agriculture and local plant-based food production can also help. Research and innovation support should be established to fund research on plant-based nutrition and alternative protein sources. Additionally, behavioral change campaigns can include promoting "default plant-based meals" in cafeterias and restaurants and strategic store placement and marketing to encourage plant-based choices.

This systematic review has several limitations, including the heterogeneity of the studies, the inclusion of only peer-reviewed, English-language studies, and the temporal bias introduced by restricting the search to studies published between 2013 and 2025. These limitations limit the ability to draw definitive quantitative conclusions and necessitate a narrative synthesis approach to summarize the findings. Despite these limitations, the comprehensive search strategy, which included multiple databases and manual reference checks, ensured a thorough identification of relevant studies, minimizing the risk of overlooking key evidence. The rigorous quality assessment process, using standardized tools like the Cochrane Risk of Bias Tool and the NOS, enhanced the reliability of the findings by minimizing bias and ensuring only methodologically sound studies were included. Adhering to PRISMA guidelines ensured a transparent and reproducible process, allowing other researchers to replicate the research or build upon its findings. The use of narrative synthesis, including thematic and comparative analyses, provided a nuanced understanding of the evidence, exploring patterns, inconsistencies, and gaps in the literature and offering valuable insights into the role of plant-based diets in preventive medicine. While this review provides valuable insights into the available research and supports the benefit of a plant-based diet in the prevention of chronic health problems, it did not include a meta-analysis to include some statistical data, which could be helpful. Thus, future studies should include a meta-analysis to supplement the findings.

## Conclusions

Plant-based diets represent a promising strategy in preventive medicine, offering significant benefits in reducing the risk of chronic diseases such as type 2 diabetes, cardiovascular diseases, obesity, and metabolic syndrome. This systematic review highlights their efficacy in improving metabolic health, weight management, cardiovascular risk, and gut microbiome composition, supported by consistent evidence across numerous studies. It showed that plant-based diets offer a comprehensive and sustainable approach to chronic disease prevention and public health improvement, with significant potential to transform dietary practices and healthcare strategies worldwide. However, the review also underscores existing challenges, including variability in diet definitions, limited long-term studies, underrepresentation of diverse populations, and the need for more rigorous mechanistic and intervention research. By addressing these gaps, future investigations can refine our understanding of plant-based diets, tailoring interventions to diverse populations and cultural contexts. Public health policies promoting plant-based diets and educating healthcare providers and individuals could enhance adoption and mitigate barriers such as nutrient deficiencies. Beyond health benefits, the environmental sustainability of plant-based diets further amplifies their relevance in addressing global health and ecological challenges. Continued research and policy efforts are essential to maximize their impact and ensure equitable health outcomes for all populations.
